# Decision-making process of Kala Azar care: results from a qualitative study carried out in disease endemic areas of Nepal

**DOI:** 10.1186/2049-9957-2-14

**Published:** 2013-07-12

**Authors:** Shiva Raj Adhikari, Siripen Supakankunti, M Mahmud Khan

**Affiliations:** 1 Department of Economics, Patan Multiple Campus, Tribhuvan University, Kathmandu, Nepal; 2 Faculty of Economics, Chulalongkorn University, Bangkok, Thailand; 3 Department of Health Services Policy and Management, University of South Carolina, Carolina, USA

**Keywords:** Kala Azar, Decision-making process, Utilization, Nepal

## Abstract

**Background:**

Analysis of consumer decision making in the health sector is a complex process of comparing feasible alternatives and evaluating the levels of satisfaction associated with the relevant options. This paper makes an attempt to understand how and why consumers make specific decisions, what motivates them to adopt a specific health intervention, and what features they find attractive in each of the options.

**Method:**

The study used a descriptive-explanatory design to analyze the factors determining the choices of healthcare providers. Information was collected through focus group discussions and in-depth interviews.

**Results:**

The results suggest that the decision making related to seeking healthcare for Kala Azar (KA) treatment is a complex, interactive process. Patients and family members follow a well-defined road map for decision making. The process of decision making starts from the recognition of healthcare needs and is then modified by a number of other factors, such as indigenous knowledge, healthcare alternatives, and available resources. Household and individual characteristics also play important roles in facilitating the process of decision making. The results from the group discussions and in-depth interviews are consistent with the idea that KA patients and family members follow the rational approach of weighing the costs against the benefits of using specific types of medical care.

**Conclusion:**

The process of decision making related to seeking healthcare follows a complex set of steps and many of the potential factors affect the decision making in a non-linear fashion. Our analysis suggests that it is possible to derive a generalized road map of the decision-making process starting from the recognition of healthcare needs, and then modifying it to show the influences of indigenous knowledge, healthcare alternatives, and available resources.

## Multilingual abstracts

Please see Additional file 1 for translations of the abstract into the six official working languages of the United Nations.

## Background

Analysis of consumer decision making in the health sector is a complex process of comparing feasible alternatives and evaluating the levels of satisfaction associated with the relevant options. Using the qualitative research method in understanding consumer behavior in health is quite intricate [1]. One significant concern is that the choice of providers, as well as the number of provider contacts, is affected by a host of variables in a non-linear fashion. Qualitative responses may not be able to capture these nonlinearities unless probed explicitly. A number of theoretical frameworks have been developed to facilitate the understanding of healthcare utilization complexities [2,3]. These models provide an understanding of an individual’s decision to utilize health care, however, they generally lack empirical corroboration. Previous models related to healthcare utilization help to understand situation and coverage of underlying factors but several deficiencies limit their ability to be generalized. It is difficult to identify stages of an individual’s decision-making process to get empirical verification of these models. Most of the models found in the literature are based on developed countries, particularly the USA [4], and it is important to examine how the preferences, context and factors affecting decision making are different for the developing world.

In this paper, we have made an attempt to understand how and why consumers make specific decisions, what motivates them to adopt a specific health intervention, and what features they find attractive with each of the options. To answer these questions, we identify a number of primary factors that influence consumers’ decision making and develop a road map to show how consumers make decisions related to the utilization of healthcare services.

Knowing how consumers decide on the choice of healthcare services and how they prefer to use various resources, such as time, money and effort, might help policy makers improve the delivery of healthcare services. The analysis of why and how people utilize health services will be helpful in providing the right type of care and to make health services more accessible to poor patients. This paper is an attempt to answer some of these questions on patient behavior. The focus of the study was to analyze the decision-making behavior of an at-risk population of Kala Azar (KA), a neglected tropical disease, and to describe how various economic factors interact with other social factors in the decision-making process. This study has adopted qualitative approaches of gathering information on KA patients. Information was obtained through in-depth interviews and focus group discussions (FGD).

## Methods

### Study design

The paper has used a descriptive-explanatory design. Information was gathered through FGD and in-depth interviews. The discussion – designed to reveal beliefs, opinions, and motives – took place in an informal setting. In-depth interviews using a semi-structured template were designed to better understand the attitudes, beliefs, and knowledge of the population in KA endemic areas of Nepal. Open-ended questions were asked to illicit information related to general knowledge about KA, health seeking behavior, choice of services, factors affecting the decision to utilize or not to utilize KA services, attitudes, beliefs, risks, healthcare cost, among others. The primary target group for discussions and information gathering of the study was the individuals with KA or caregivers of KA patients. We will use the term “KA group” to refer to this group of individuals in the community.

### Study site and sampling procedures

KA (visceral leishmaniasis), a tropical disease (also known as a disease of the poor), is usually fatal, if not treated. KA threatens almost one quarter of Nepal’s population, and is confined to 12 districts in the country. This study was conducted in all of the KA endemic districts in Nepal. The multistage convenience sampling approach was used to select the communities and the participants. Eight communities with high KA incidence rates were chosen for the purpose of this study. Social mapping, an activity to locate KA experienced households in relation to other households, was used at each of the research sites to identify the highest KA incidence neighborhoods. Following this, eight to 12 participants, male and female but not always in equal numbers, were selected for conducting the FGDs, and then 2 or 3 participants were purposely selected from each group for in-depth interviews. The choice of individuals for in-depth interviews was guided by the ability of the persons to provide additional information on the disease, and the decision-making process of patient’s families and/or community leaders or representatives. Research methods included the use of participatory analysis tools and FGD with the KA group. A total of 101 people were consulted for the qualitative study. No incentives were offered for participation in the study.

### Data collection

A research team consisting of three individuals (two men and a woman; an economist, sociologist, and a medical worker from a health institution) conducted the study. All were trained in their specific disciplines and had prior field-level research experience. Training sessions were conducted about the specific goals, structures, timeframes, and procedures of the study. Among the three researchers, one was trained to be the moderator and the other two were trained as transcribers. Standard guidelines, as proposed by Stewart and Shatmdasani [5] and Ritchie and Lewis [6], were followed during the data collection process. The team spent two days in each community. The research team explained the purpose of the study to the formal leaders of the communities and then to the KA group members. The team members ensured an open and friendly environment to encourage the participants to express their opinions without any hesitation. FGDs were conducted in a non-directive manner, however, discussions were used as a source of new and fresh ideas to develop new hypotheses. The FGDs, thus, probed in-depth specific aspects on how people allocate their resources, and how they decide about healthcare seeking and types of services to use. Before conducting the FGDs and the in-depth interviews, some background information on the selected communities was obtained from primary and secondary sources. In some group meetings, the participants were requested to do an exercise to obtain a few of the quantitative parameters like the proportion of poor people in the community, the proportion of KA patients using different types of medical care services, amongst others, through a process of consultation and consensus.

Methodological triangulation was employed to improve the accuracy of the information obtained. This is done through the use of various approaches, such as team composition, secondary information, and discussions with participants with different perspectives. FGDs and in-depth interviews with the same participants were conducted in order to increase convergent validity. Debriefing was conducted immediately after the FDG while the team was still in the field so that the team can evaluate the quality of the session conducted. This also allowed the team to improve their skills and to crosscheck the responses. We used causal-impact analysis, or flow diagrams, to show the links between different underlying factors of the health seeking decision-making process and the flow of events.

### Data analysis

Coding of the FDGs, ethnographic field notes, information collected from the in-depth interviews, and other relevant documents were used for analysis of emerging themes and presentation of data in the form of narratives. Final themes were generated through discussions between the authors on the basis of their independent initial analysis. Data analysis was an iterative process, whereby themes were continuously generated, revised, and re-examined.

## Results

### Summary of data in words

During the discussion, the participants explained their own perception about KA and the risk of KA in the community:

“*Kala Azar is a very dangerous disease* … *Last year many people were killed by it*. *When I became sick with a high fever*, *I was afraid and went directly to the hospital*…” (Male, community 1)

“*The community is a KA*-*prone area where dozens of people get infected every year*.” (Female, community 4)

“*KA treatment is not new for me*. *The last one was the second attack for me*… *I went directly to the public hospital last time*.” (Female, community 7)

People are aware of the risk of KA, probably because it is a fatal disease with clear symptoms and progression. It appears that people in the community know the symptoms of the disease quite well. Perception about the disease and subjective evaluation of urgency for seeking care are the principal driving forces triggering the first visit to health professionals. After the first contact, however, it is the individual's and the professional's notions of need that drive the demand for additional care, such as paramedical tests, use of drugs, follow ups, consultations with another provider, amongst others. The change in the individual's perception of healthcare need and efficacy of the earlier visits affect subsequent visits or visits to different providers.

*“I visited the private clinic in Lahan.... A doctor gave me some medicines and suggested thatI come back after seven days*… *my health was going down*, *I went back again before the week was up*… *I did a blood test*… *my problem did not improve*…*it became even worse*…*then I decided to go to the Jilla Aspatal* (*district hospital*).” (Male, community 6)

“ …*First time*, *I went to the drugstore to buy the drugs*, *but they did not help*…*my brother*-*in*-*law suggested that I went to private clinic in India*… *bhukhar* (*high fever*) *was still continuing*… *I went to a Dhami* (*a traditional healer*)…*again*, *I went to the primary health center*, [*and*] *the doctor suggested that I went to the Jilla Aspatal*…*after the treatment at Jilla Aspatal*, *I recovered*.” (Female, community 5)

In Nepal, effective KA care is available only in the public hospital; therefore, the choice of provider is the most important determinant of cure. Public (district and zonal) hospitals in the KA endemic areas provide KA care free of charge. At first, it was unclear why people chose other healthcare providers, as the KA services are completely free at the public hospitals. Upon probing, this issue became somewhat clear:

“*Rich people can pay for transportation cost*, *food cost*, *and other things*… *They can stay in a hotel for few days if it is necessary to stay in the area while getting treatment at the district hospitals*…. ” (Female, community 2)

The hospitals are located in the urban areas of the districts and the KA patients have to spend a significant amount of time, effort, and other resources to obtain care from the hospital.

“*I went to the aushadhi pasal* (drugstore) *near the village*. *I was given some medicine*, *but the medicine did not work*… *we didn*’*t have the money to buy more medicine*… *When my condition became serious*, *my wife borrowed 3000 rupees with a 5*% *interest rate per month*.” (Male, community 8)

“*I cannot go to the hospital alone*. *My husband needs to go to work*…[*so*] *I stayed home*.” (Female, community 3)

“*If we wanted to go to the hospital*, *we needed to go in the morning and could expect to come back in the evening*. *I would not be able to work and would not get my Rs 160*. *So*, *after work*, *I went to the pharmacy* – *this was easy for me and cost only about Rs 75*.” (Male, community 5)

“*It is often easier* (*convenient*) *to go to a private place close by than going to the Jilla Aspatal*…*there is usually a long queue there*.” (Male, community 1)

“*Some services are free*, *but if we are hungry*, *we need to eat something and that is not free*, *bus fare is not free*, *accommodation is not free*…*an urban area is an urban area* …*everything there is more expensive*.” (Female, community 2)

“*My wife and I went to the Jilla Aspatal on foot to save bus bhada* (*transportation cost*). *It took us three and a half hours*.” (Male, community 6)

“My brother took me on a bicycle to the hospital; it took us three hours to get there.”

(Male, community 7)

Therefore, although the KA services are provided free of charge, patients and the accompanying family members have to pay for transportation, accommodation, and food. Therefore, the expected cost of treatment turns out to be high for them.

“…I got ill. I could not work for more than a month. I had to borrow money to buy rice. If I used that money to go to the hospital, my kids would have gone hungry…” (Male, community 4)

“*My wife went to the thula sahu* (*local landlord*) *to ask for some money*. *He told her that she can borrow money at the monthly interest rate of 7*%. *With this high interest rate*, *it will be difficult for us to repay the principal and the interest*. *We decided not to borrow the money and decided not to go to the hospital*.” (Male, community 8)

“*She had been in bed for more than three weeks*. *I was worried about her health and decided to borrow 2000 rupees*…*for her treatment at the hospital*.” (Male, community 6)

“*Now*, *we are facing a greater state of poverty than before because I cannot work in the field due to physical weakness*.” (Male, community 3)

“*Those KA patients who are from very poor families cannot afford the treatment and quietly accept the illness as their destiny and die from it due to lack of treatment*.” (Female, community 4)

“*In the course of my treatment*, *I had to sell my one Kattha land and became landless again*.” (Male, community 5)

The quantitative parameter derivation process indicates that almost two fifths of individuals with KA preferred self-care or home care in KA endemic areas. One of the major reasons for choosing self-care is the lack of financial resources for seeking modern health care. Distance to the facilities, travel time, and waiting time are also important in affecting the utilization of modern health facilities. Individual and household characteristics are also important in the decision-making processes on the choice of healthcare providers.

“I was still worried about my wife. She had already undergone KA treatment three *times* – *once in the hospital in India for 15 days*, *again for seven days in the nursing home*, *and 18 days in public hospitals*.” (Male, community 1)

“*Wage labor is the main source of livelihood for us*. *We have two kids*. *Both of us*, *husband and wife*, *work as agricultural laborers*, *each earning Rs*. *100 per day*. *I cannot go with her to the hospital as her caretaker*… *It is not possible for us to survive if both breadwinners are away from work*.” (Male, community 4)

“*We have only one child*, *when he became sick*…*next day*, *we went to the hospital*.” (Female, community 3)

“*My father*, *who is 61 years old*, *did not want to go the hospital*…*he wanted to buy drugs from the drugstore*.” (Male, community 7)

“*My wife preferred to take rest* (*self*-*care*) *at her parents*’ *home when she became ill with KA*…. *It was very difficult to convince her to go to the hospital*.” (Male, community 4)

In general, most of the participants of the discussion groups felt that individuals who become sick with KA usually consulted a healthcare provider within one week after contracting the disease. According to the group members, about two-fifths of patients choose home care or self- care for the treatment of KA.

Self-care refers to healthcare practices that individuals initiate or perform on their own for restoring health. The self-care activities vary widely, from resting to restoring physical energy, to seeking advice from a local individual or family member for alleviating discomfort or pain, to taking locally available herbs and using water therapy, to simply doing nothing. Self-care may appear to be low cost or free but the activities do take a significant amount of time and effort. About a quarter of KA patients probably consult the drugstore personnel as the first contact provider. The subjective opinion of the group members was that about four out of five KA affected households need to borrow money to pay the costs associated with KA treatment. In general, the rates of interest charged by lenders were very high, usually more than 60% per year.

### Access to KA treatment services

Nepal’s public healthcare system, if hierarchically structured, could be compared to a four-layered pyramid: primary care at the below district level, primary care at the district level, and secondary care and tertiary care, from bottom to top level, respectively. Apart from the public system, there has been a significant growth in the private sector in providing healthcare services. KA treatment and diagnosis services are available at the (public) district hospitals. A recent national representative survey suggested that more than 50% of rural people had access to district hospitals within a one-hour travel time, whereas more than 80% of rural people have access to below the district level primary care within a one-hour travel time [7]. There is a referral system from bottom to top level health care. In terms of financial access to KA care, KA related treatment, drugs, and diagnostic services are provided free of charge at the district hospital. KA treatment services are not available in the private hospitals.

### Analysis of decision-making processes

Interpreting the process of decision making based on a limited number of statements or categorization of statements may not be sufficient, especially because healthcare seeking is affected by complex interactions among many individual, household, community, and healthcare supply variables. We have analyzed the data through an iterative process that is continuously generating, revising, and re-examining the themes.

The detail discussions and probing with the participants indicate that five groups of variables affect the final decision-making process on health seeking. These five groups are: healthcare need related factors, indigenous knowledge, choice of healthcare providers in the locality, resources, and characteristics of individuals and households although these categorizations are not mutually exclusive. Seeking treatment for KA is conditional on illness and therefore, recognition of the presence of illness is the first trigger of the decision-making process. Age, sex, pain and suffering, physical weakness, and signs and symptoms of KA determine the health status of individuals, as well as indicating the degree of severity of the illness.

**Figure 1:**
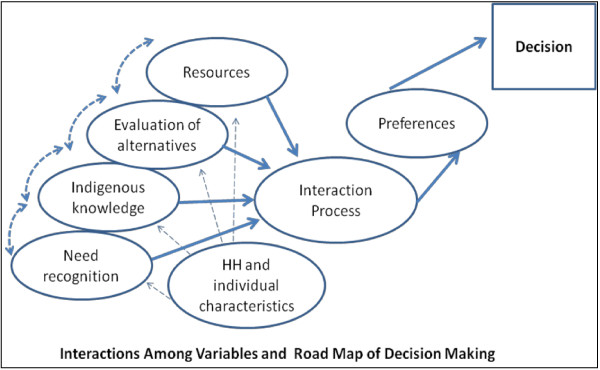
**The decision**-**making process of KA patients as indicated by participants in the qualitative surveys. **Note: The ovals show the decision-making process at each level and the rectangle shows the final decision. The causal relationships are shown by solid arrows, two-way interactive relationships by dashed two-side arrows, and one-way interactive relationships by dashed one-side arrows.

Once the patients and the family members become convinced that the illness is becoming more severe, the need for medical care is created. Need for medical care does not necessarily translate into taking concrete steps for seeking medical attention. The gap between the need for care and medical care seeking is related to the cost of obtaining medical care services, perceived benefit of the care, affordability of the health services, willingness to scarify something to meet the healthcare need, amongst others. Once need (health status) recognition occurs, consumers start searching for information on sources and types of care. The set of information obtained from past experiences, experiences of family members and friends, religious beliefs, and attitudes can be defined as indigenous knowledge. Based on indigenous knowledge and new knowledge about treatment, patients construct and evaluate available healthcare alternatives. The alternatives are evaluated on the basis of resource needs associated with the utilization of the alternative resources and the resources the household has. This evaluation lets the patients decide where to get the medical care services from, when to seek care, what to consume, and what to utilize and not to utilize. The decision-making process is illustrated in Figure 1.

The preference structure is the most important aspect that ultimately determines whether the households will seek care from outside sources for the KA patients. In this case, central to the choice problem, is the allocation of three precious resources: money, time, and effort. Households collect information on the expected cost of care, time inputs needed for seeking care, travel cost, and other costs related to healthcare seeking. Financial resources appear to be the most important constraint faced by KA patients and most KA patients need to borrow money to obtain care from an outside source.

There might be some conflict between convenience (place, time, and credit) and the quality of services. For example, use of home-based care is clearly more convenient, but the quality of this care would be very low. To increase the satisfaction of the household members, households choose the right mix of convenience and expected quality of services. Convenience can be approximated by calculating the total cost of obtaining care from a provider, including the opportunity costs of time and transportation. The higher the cost, the lower the degree of convenience will be. Quality of care is related to expected health improvement. Thus, the selection of the healthcare provider is determined by the interaction between convenience (costs) and the self-assessed health outcomes after the utilization of the service. If severity increases, the individual is more likely to choose better quality services.

## Discussion

A considerable amount of quantitative research has been conducted to understand the healthcare seeking behaviors of patients [8,9]. Qualitative studies are rarely used to examine the decision-making processes related to healthcare demand and utilization [10,11]. In spite of its significant contribution towards better understanding of demand patterns, quantitative analysis alone may not be sufficient to capture all the aspects of complex relationships among the underlying factors. Some sociological studies suggest that socioeconomic variables, demographic, and access and need of health related factors determine the healthcare utilization decisions [12-15].

In this qualitative study, we found that healthcare need, indigenous knowledge, available healthcare alternatives, and resources jointly determine decision making related to health seeking from outside healthcare providers. Andersen’s model [2] was again revised to form three components with a linear relationship: primary determinants (population characteristics, healthcare system, external environment); health behavior (personal health characteristics, use of health services); and health outcomes (perceived health status, evaluated health status, consumer satisfaction). But this study also found that a nonlinear relationship and interaction between factors in the decision process of healthcare utilization exists. Previous models of decision-making behavior to utilize health care are derived from speculations and assumptions that are theoretically sound, but lacking empirical support and subject to limitations [16]. We develop the model for decision-making behavior to utilize the health care from empirical analysis.

We have explored the road map of the decision-making process. Through our discussions with the participants, it became clear that the participants viewed the decision-making process at each stage as an interactive process. Due to the complexities involved, it was not possible to present all the aspects of perceived decision making in a simple diagram. It is clear, however, that the process defined at each stage represents a rational process of decision making based on the experiences, cultural values, opinions and ideas, priorities, and preferences of patients and their families in the community.

Health policy is able to change the behavior of patients in at least two ways: through the provision of a greater degree of choices and by lowering the financial constraints. Providing KA treatment services free of charge is not sufficient to lower the financial constraint enough for poor households. This is because transportation and other associated costs are relatively high and poor KA patients find it difficult to find the money to afford a trip to district hospitals. Another possible way to improve access would be to make the KA treatment services available at lower level facilities. Demand side financing is another approach of encouraging patients to seek appropriate care.

To generalize the findings of the qualitative study, it is important to compare the results with the results of a quantitative study. The qualitative study has a number of limitations as well. For example, it is possible that moderator biases are present in our qualitative study. The research team spent only two days in each of the communities for the collection of information, and such a short stay may not be adequate to understand the preferences and perceptions of the rural population.

## Conclusion

The process of decision making related to healthcare seeking follows a complex set of steps and many potential factors affect the decision making in a non-linear fashion. Our analysis suggests that it is possible to derive a generalized road map of the decision-making process starting from the recognition of healthcare needs, and modifying it to show the influences of indigenous knowledge, healthcare alternatives, and available resources. The group discussions imply that people are rational and they compare costs and benefits based on their perception, experiences, priority, and preferences. In most of the discussions, the participants emphasized the importance of resource availability, and the availability of money and time against the demand for medical care services. Many poor households must borrow money at relatively high interest rates to finance the out-of-pocket costs associated with seeking care from public hospital and clinics. Introducing demand side financing and increasing choices for KA care should be considered in order to encourage KA patients to seek effective treatment.

### Ethical approval

Ethical clearance and approval was obtained from the Nepal Health Research Council, Kathmandu and the World Health Organization HQ, Geneva. Written consent was obtained from all individuals participating in the study.

## Competing interest

The authors wish to declare that they have no competing interests.

## Authors’ contributions

SRA and SS designed the study protocol; SRA conducted the field survey; SRA, SS, and MK carried out the data analysis and interpreted the data; and SRA and MK drafted the manuscript. All authors read and approved the final manuscript. SRA and MK are guarantors of the paper.

## Supplementary Material

Additional file 1 Multilingual abstracts in the six official working languages of the United Nations. Click here for file
