# Supplementary material for: Decision-making process of Kala Azar care: results from a qualitative study carried out in disease endemic areas of Nepal
Source: Infect Dis Poverty. 2013 Jul 12;2:14. doi: 10.1186/2049-9957-2-14 (PMC3717077; doi:10.1186/2049-9957-2-14)
Supplement: Additional file 1 — Multilingual abstracts in the six official working languages of the United Nations. [file 2049-9957-2-14-S1.pdf]

Translation of the abstract into the six official working languages of the United Nations

عملية اتخاذ القرار المتعلقة بالعناية بمرض الكالا – آزار: نتائج دراسة نوعيّة أجريت في مناطق موبوءة بالمرض في نيبال

شيفا راج أديكاري، سيريبين سوباكنكونتي، م محمود خان

الملخّص

الخلفية إن تحليل قرارات المستهلك في القطاع الصحي عملية معقدة تنطوي على مقارنة البدائل الممكنة وتقييم مستويات الرضا المرتبطة بالخيارات ذات الصلة. في هذا البحث محاولة لفهم كيف ولماذا يقدم المستهلكون على اتخاذ قرارات معينة، ما يحفزهم على تبني تدخلات صحية معينة، وما هي الميزات التي يجدونها جذابة في كل من الخيارات.

الطريقة استخدمت هذه الدراسة تصميما وصفيا مفسرا من أجل تحليل العوامل المحددة لاختيارات مقدمي الرعاية الصحية. وتم جمع المعلومات عن طريق نقاشات المجموعات المركزة واللقاءات المعمقة.

النتائج تشير النتائج إلى أن اتخاذ القرار المتعلق بطلب الرعاية الصحية لعلاج الكالا آزار هو عملية تفاعلية معقدة. يتبع المرضى وأفراد أسرهم خريطة طريق محددة بشكل جيد من أجل اتخاذ القرار. تبدأ عملية اتخاذ القرار بالتعرف على احتياجات الرعاية الصحية ثم يجري تعديلها بسبب عدد من العوامل، كالإدراك الفطري، البدائل العلاجية، والموارد المتاحة. كما تلعب الخصائص المنزلية والفردية أيضا دورا هاما في تسهيل عملية صنع القرار. تتسق نتائج المناقشات الجماعية والمقابلات المتعمقة مع فكرة اتباع مرضى الكالا آزار وأفراد أسرهم لنهج منطقي للمفاضلة بين تكلفة وفوائد استخدام أنواع معينة من الرعاية الطبية.

الاستنتاج تتبع عملية اتخاذ القرار المتعلقة بطلب الرعاية الصحية مجموعة معقدة من الخطوات وتؤثر عدة عوامل محتملة على صنع القرار بطريقة غير خطية. يشير تحليلنا إلى أنه من الممكن وضع خريطة طريق عامة لعملية صنع القرار ابتداء من الاعتراف بالحاجة للرعاية الصحية، ومن ثم تعديلها لإظهار تأثيرات الإدراك الفطري، بدائل الرعاية الصحية، والموارد المتاحة.

Translated from English version into Arabic by Lina SM, through

黑热病护理的决策过程：在尼泊尔流行区的定性研究

Shiva Raj Adhikari, Siripen Supakankunti, M Mahmud Khan

摘要

引言　在卫生部门消费者决策分析是一个复杂的过程，需比较可行的替代方式，并评估相应选项的满意程度。本文试图了解消费者如何以及为什么作出决定，是什么促使他们采取特定的健康干预，对他们而言有吸引力的选项有什么特点。

方法　该研究使用描述性说明设计，对确定医疗服务提供者选择的因素进行分析。采用焦点小组讨论和深入访谈的方式收集资料。

结果　结果表明，确定黑热病治疗方案的决策过程是一个复杂的、互动的过程。患者和家属在作决策时遵循一个明确的路线图。决策过程是先确认医疗保健需求，然后根据一些其他因素进行修改（如本土知识、医疗保健替代品、可用资源）。在促进决策的过程中，家庭和个人的特点也发挥着重要作用。小组讨论和深入访谈的结果与黑热病患者及其家属的想法一致，即按照合理的方法权衡使用特定类型医疗保健的成本。

结论　确定具体医疗方案的决策过程是按照一系列复杂步骤进行的，许多潜在因素以非线性的方式影响决策。我们的分析表明，可以得出一个决策过程的整体路线图，即先确认医疗保健需求，然后根据本土知识、医疗保健替代品和可用资源的影响进行修改。

Translated from English version into Chinese by Yang Pin, through

CDC CHINESE CENTER FOR DISEASE CONTROL AND PREVENTION
NATIONAL INSTITUTE FOR PARASITIC DISEASES

Processus décisionnel des Soins Kala Azar : Résultats d'une étude qualitative réalisée dans les zones endémiques de la maladie au Népal

Par Shiva Raj Adhikari, Siripen Supakankunti, M Mahmud Khan

Synthèse

Antécédents L´analyse de la prise de décisions du consommateur dans le secteur de la santé est un processus complexe qui consiste à comparer des alternatives réalisables et à évaluer les niveaux de satisfaction associée aux options pertinentes. Cet article tente de comprendre comment et pourquoi les consommateurs adoptent des décisions précises, quels motifs les portent à adopter une intervention sanitaire spécifique, et quelles sont les caractéristiques qu'ils trouvent attrayantes dans chacune des options.

Méthode L'étude a utilisé une conception descriptive et explicative pour analyser les facteurs qui déterminent le choix des prestataires de soins. Les renseignements ont été recueillis par le biais de groupes de discussion restreinte et d'entretiens approfondis.

Résultats Les résultats suggèrent que la prise de décisions liées aux soins de santé recherchés pour le traitement de Kala Azar (KA) est un processus interactif complexe. Les patients et les membres de la famille suivent une feuille de route bien définie pour la prise de décisions. Le processus décisionnel commence à partir de la reconnaissance des besoins en soins de santé et est ensuite modifié par un certain nombre d'autres facteurs, tels que les savoirs autochtones, les alternatives de soins de santé et les ressources disponibles. Les caractéristiques individuelles et celles de ménage jouent aussi un rôle important dans le processus décisionnel ainsi facilité. Les résultats issus des groupes de discussion et des entretiens approfondis sont conformes avec l'idée que les patients de KA ainsi que les membres de leur famille suivent l'approche rationnelle d'évaluer les coûts par rapport aux avantages de l'utilisation des soins médicaux particuliers.

Conclusion Le processus décisionnel lié aux soins de santé recherchés fait suite à un ensemble complexe de mesures, et de nombreux facteurs potentiels affectent la prise de décisions de manière non linéaire. Notre analyse suggère qu'il est possible de tirer une feuille de route généralisée du processus décisionnel à partir de la reconnaissance des besoins de soins de santé et de le modifier ensuite pour indiquer l'influence des savoirs, les alternatives de soins de santé et les ressources disponibles.

Translated from English version into French by MultiPro, through

TRANSLATORS
WITHOUT BORDERS

Процесс принятия решения при лечении индийского висцерального лейшманиоза (лихорадки «дум-дум»): результаты качественного исследования, проведенного в зараженных районах Непала

Шива Радж Адхикари, Сирипен Супаканкунти, М. Махмуд Хан

Резюме

История вопроса Анализ процесса принятия решений потребителем в секторе здравоохранения представляет собой сложную задачу, предполагающую сравнение осуществимых альтернатив и оценку уровней удовлетворенности по соответствующим вариантам. Настоящее исследование – это попытка понять, как и почему потребители принимают те или иные решения, чем руководствуются при выборе определенного вида медицинской помощи и какие характеристики являются привлекательными в каждом из вариантов.

Методология В рамках исследования используется описательно-объяснительный подход к анализу факторов, определяющих выбор поставщиков медицинских услуг. Сбор данных осуществлялся посредством проведения обсуждения в фокус-группах и подробных интервью.

Результаты Результаты показывают, что принятие решения при выборе способа лечения индийского висцерального лейшманиоза представляет собой сложный и многогранный процесс. Пациенты и их родственники придерживаются четкой схемы выбора нужной альтернативы. Процесс принятия решения начинается с признания необходимости медицинской помощи и испытывает на себе воздействие целого ряда иных факторов, включая инстинктивное знание, альтернативные виды медицинского обслуживания и доступные ресурсы. Бытовые условия и индивидуальные черты характера также играют важную роль в процесс принятия решений. Результаты, полученные в рамках групповых обсуждений и подробных интервью, лишь подтверждают идею о том, что больные лихорадкой «дум-дум» и их родственники предпочитают рациональный подход уравновешивания издержек и потенциальной пользы отдельных видов медицинского обслуживания.

Заключение Процесс принятия решений в сфере здравоохранения состоит из нескольких сложных шагов и испытывает на себе влияние нескольких косвенных

факторов. Наш анализ показал, что вполне возможно создать общую схему принятий решений, начиная с признания необходимости медицинского обслуживания и заканчивая ее постепенной модификацией под влиянием инстинктивного знания, альтернативных видов медицинского обслуживания и доступных ресурсов.

Translated from English version into Russian by Irina Zayonchkovskaya, through

TRANSLATORS
WITHOUT BORDERS

Proceso de toma de decisiones en el cuidado de la leishmaniosis visceral: resultados de un estudio cualitativo realizado en zonas endémicas de la enfermedad en Nepal

Shiva Raj Adhikari, Siripen Supakankunti, M Mahmud Khan

Resumen

Antecedentes El análisis de la toma de decisiones del consumidor en el sector salud es un proceso complejo que consiste en comparar las alternativas viables y evaluar los niveles de satisfacción asociados con las opciones correspondientes. Este trabajo intenta entender cómo y por qué los consumidores toman decisiones específicas, qué los motiva a adoptar una intervención específica de salud y qué características encuentran atractivas en cada una de las opciones.

Método En el estudio se utilizó un diseño descriptivo-explicativo para analizar los factores que determinan las elecciones de los proveedores de salud. La información se recogió a través de discusiones de grupos focales y entrevistas en profundidad.

Resultados Los resultados sugieren que la toma de decisiones relacionada con la búsqueda de atención médica para el tratamiento de leishmaniosis visceral (LV) o kala-azar es un proceso complejo e interactivo, donde los pacientes y sus familiares siguen una hoja de ruta bien definida para la toma de decisiones. El proceso de toma de decisiones comienza desde el reconocimiento de las necesidades de atención médica y luego, lo modifican diferentes factores tales como: los conocimientos indígenas, las alternativas de salud y los recursos disponibles. Las características individuales y del hogar también juegan un papel importante en la facilitación del proceso de toma de decisiones. Los resultados de las discusiones de grupos y las entrevistas en profundidad son consistentes con la idea de que los pacientes con LV y sus familiares siguen el enfoque racional de sopesar los costos y beneficios del uso de determinados tipos de atención médica.

Conclusión El proceso de toma de decisiones relacionado con la búsqueda de atención médica sigue una serie de pasos complejos y muchos de los factores potenciales afectan la toma de decisiones de una manera no lineal. Nuestro análisis sugiere que es posible derivar una hoja de ruta generalizada del proceso de toma de decisiones comenzando desde el reconocimiento de las necesidades de atención médica y luego, modificándola para mostrar

las influencias de los conocimientos indígenas, las alternativas de la salud y los recursos disponibles.

Translated from English version into Spanish by Natalia Guzmán, through

TRANSLATORS
WITHOUT BORDERS
